# Microbial production of melanin and its various applications

**DOI:** 10.1007/s11274-020-02941-z

**Published:** 2020-10-12

**Authors:** Anh N. Tran-Ly, Carolina Reyes, Francis W. M. R. Schwarze, Javier Ribera

**Affiliations:** 1 Laboratory for Cellulose & Wood Materials, Empa, 9014 St. Gallen, Switzerland; 2 Department of Civil, Environmental and Geomatic Engineering, ETH Zürich, 8093 Zürich, Switzerland

**Keywords:** Fungi, Microbial melanin, Pigments, Upscaling

## Abstract

Melanins are natural biopolymers that are known to contribute to different biological processes and to protect organisms from adverse environmental conditions. During the past decade, melanins have attracted increasing attention for their use in organic semiconductors and bioelectronics, drug delivery, photoprotection and environmental bioremediation. Although considerable advances in these fields have been achieved, real-world applications of melanins are still scarce, probably due to the limited and expensive source of natural melanin. Nevertheless, recent biotechnological advances have allowed for relatively large-scale production of microbial melanins, which could replace current commercial melanin. In this review, we first describe different melanin sources and highlight the advantages and disadvantages of each production method. Our focus is on the microbial synthesis of melanins, including the methodology and mechanism of melanin formation. Applications of microbial melanins are also discussed, and an outlook on how to push the field forward is discussed.

## Introduction

Melanin is an ancient pigment that occurred very early in all living organisms (Zhang et al. 2010; Glass et al. 2012). Melanin is typically known for its unique ability to absorb a wide range of radiations (Brenner and Hearing 2008; Liu et al. 2013). Moreover, melanization is considered a survival strategy for many organisms inhabiting unfavorable environmental conditions. Owing to the multifunctionality of the pigment, it has been known to serve as: (a) an antioxidant and radical scavenger (Ju et al. 2011; Le Na et al. 2019), (b) a photo-protector that efficiently absorbs and dissipates solar radiation in the form of heat (d’Ischia et al. 2015), (c) an absorber that chelates metals and binds organic compounds (Karlsson and Lindquist 2016; Tran-Ly et al. 2020) and (d) an organic semiconductor (Bothma et al. 2008). Besides these functions, melanin is considered eco-friendly and biocompatible since it is naturally synthesized by most organisms. Melanin has recently burst onto the scene of materials science and green technology as a functional additive or coating that can substantially improve the performance of conventional materials for different applications. However, upscaling production and extraction protocols of melanin needs further optimization so that it can be used for developing novel materials.

In this review, we first introduce the current understanding of melanin, along with its chemical structures and physical properties. We then present the strategies of melanin production, including the chemical synthesis and methods based on natural resources with emphasis on promising biotechnology processes using microorganisms. We highlight several recent applications of microbial melanins, and provide our perspectives on how to bring melanin closer to practical applications in materials science.

## Melanin pigments

Recent studies suggest that melanin is in fact a general term for a group of heterogeneous pigments produced by organisms of all domains of life from bacteria to mammals (the plural form “melanins” is occasionally used in sections below indicating the heterogeneous nature of melanin). In humans, melanin is the prominent pigment responsible for the colour of skin, hair and eyes (Solano 2014; d’Ischia et al. 2015). As melanin usually appears black or dark brown, the pigment derives its name “melanin” from “melanos”—an ancient Greek word for black (Borovanskỳ and Riley 2011). However, there are other pigments in this group that produce reddish or yellowish colours such as the pheomelanin found in red hair, freckles, and feathers.

Melanin has a relatively diverse and heterogeneous structure. This is due to the ubiquitous sources of melanin, which leads to its heterogeneity in composition, size, color and function. Moreover, the physicochemical properties of melanin (a highly negative charge, high molecular weight and hydrophobic nature) hinder analytical approaches to identify and characterize its structure (Pralea et al. 2019). Additionally, the pigment is insoluble in most solvents and is resistant to chemical degradation (Nosanchuk et al. 2015; Pralea et al. 2019). Chemical treatments, such as using a strong base, can be used to dissolve melanin but often alter its native structure and may even break the initial polymer into fragments. Enzymatic digestion is relatively inefficient in eliminating the protein and lipid content of natural samples (Pralea et al. 2019).

A widespread definition of melanin is “a heterogeneous polymer derived from the oxidation of phenolic or indolic compounds and subsequent polymerization of intermediate phenols and their resulting quinones” (Solano 2014). Melanin pigments can be categorized based on their chemical structures, namely, eumelanin, pheomelanin, neuromelanin and allomelanin (d’Ischia et al. 2013). Eumelanin is the black-to-brown subgroup of melanin formed by oxidative polymerization of tyrosine derivatives such as L-3,4-dihydroxyphenylalanine (L-Dopa), and it is the most common melanin found in animals, including humans (Solano 2014). Eumelanin is, therefore, by far the most relevant source from a biological and technological perspective and has been widely studied and used as a model for synthetic melanin. Pheomelanin is another type of animal melanin, found in red hair, freckles or feathers, which differs from eumelanin by the presence of sulfur in the composition since its precursor is 5-cysteinyl-Dopa. Neuromelanin is explicitly produced within human neurons by the oxidation of dopamine and other catecholamine precursors. In plants, fungi and bacteria, the identified melanin is called allomelanin. This group encompasses a variety of non-nitrogenous subgroups of melanin derived from different catecholic and dihydroxynaphtalene precursors, which are usually mentioned as catechol melanin (in plants), DHN-melanin and pyomelanin (in bacteria and fungi). Lastly, it is notable that many microorganisms can produce different types of melanin, including eumelanin via a similar pathway with mammalian melanin synthesis (Eisenman and Casadevall 2012; Cordero and Casadevall 2017) (Table 1).

**Table 1:** Summary of common melanins, sources and their corresponding precursors

Type of melanin	Producing sources	Melanin precursor
Eumelanin (DOPA-melanin)	Animals, bacteria, fungi	Tyrosine or L-Dopa
Pheomelanin	Animals	5-S-cys-Dopa
Neuromelanin	Human (brain)	Dopamine and 5-S-cys-dopamine
Catechol-melanin	Plants	Catechol
DHN-melanin	Fungi, bacteria	1,8-dihydroxynaphthalene (DHN)
Pyomelanin	Fungi, bacteria	Homogentisic acid

## Melanin isolation from conventional natural sources

**Fig. 1:**
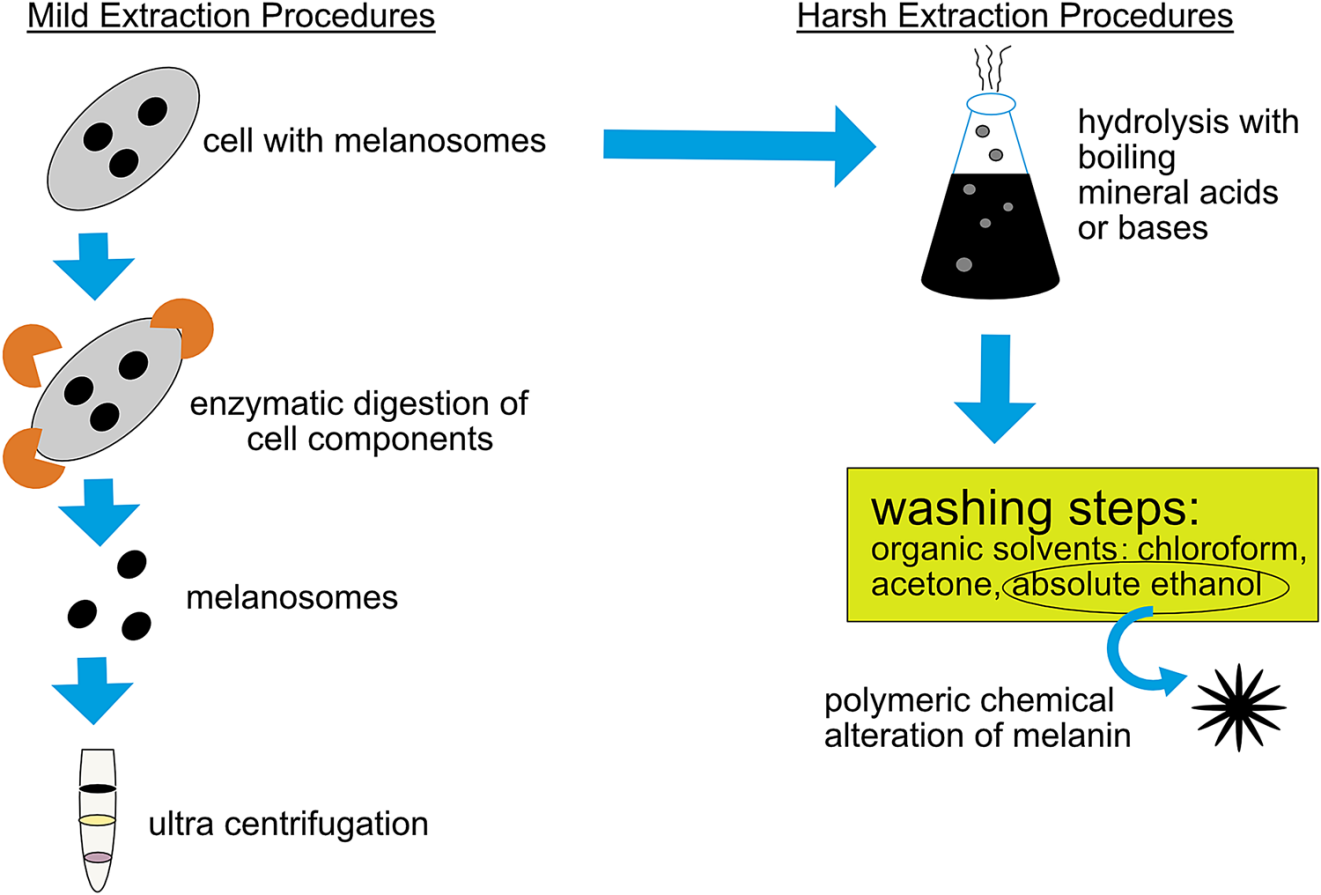
Mild and harsh melanin extraction steps currently used to obtain melanin from living cells

Conventionally, melanin is extracted from sepia ink or animals’ dark hair/feathers. One of the challenges for melanin production and extraction from these sources is that most melanins are formed inside melanosomes and are tightly bound to some cellular components such as proteins or minerals (Prota 1995). Therefore, the isolation procedure of melanin usually involves harsh chemical treatments to remove the entire protein fraction, cell debris and unconsumed nutrients. Normally, these treatments include extensive hydrolysis with boiling mineral acids or bases followed by successive washing steps with organic solvents such as chloroform, acetone or absolute ethanol (Liu and Simon 2003; Pralea et al. 2019). However, during the latter process, the melanin polymeric skeleton suffers chemical alterations (Pralea et al. 2019). Alternative strategies reported in the literature have described the use of milder isolation methodologies such as: mechanical separation using ultracentrifugation; proteolytic digestion using enzymes to eliminate the residue protein matrix; or a combination of both strategies (Novellino et al. 2000; Xiao et al. 2018) (Fig. 1). Some studies have shown that enzymatic extraction methods can retain the melanin structure and its morphology in the form of intact melanosomes better than the acid/base extraction protocols (Liu et al. 2003).

Natural melanins are complete polymers with limited potential for modification. Besides, the final drying method can have a great influence on the physical properties of melanin such as the aggregation, the surface area-to-mass ratio, and porosity (d’Ischia et al. 2013). This source-dependency makes natural melanin supply difficult and expensive for up-scaling and can result in contamination depending on its source. For example, melanin extracted from feathers of birds or the ink sac of sepia may have an increased amount of associated toxic metals related to their environmental exposure. Moreover, these melanin sources are of ethical concern as the animals, from which melanin is extracted, may need to be killed. All these factors emphasize the cautious use of natural melanin for applied research.

## Melanin production by chemical synthesis

In the last decade, the synthesis of materials with properties mimicking that of natural melanins has been extensively investigated (Lee et al. 2007; Liu et al. 2013; D’Ischia et al. 2014; Solano 2017). In chemical synthesis, polydopamine, which shares some properties with natural melanin due to their similar functional groups such as catechol, amine and imine groups (Solano 2017), is synthesized via oxidative polymerization of dopamine. The high tunability of polydopamine has rapidly promoted research on this material (Liu et al. 2014). Notably, when studies on synthetic melanin-based materials are cited, they usually refer to polydopamine and its derivatives.

Three common approaches for the synthesis of polydopamine are: (1) solution oxidation, (2) enzymatic oxidation, and (3) electropolymerization (Liu et al. 2014). Solution oxidation under alkaline conditions is widely used and involves the oxidation with oxygen and self-polymerization of the dopamine monomers. The second approach is often related to the enzymatic oxidation of L-tyrosine using the enzyme tyrosinase. Another method in this approach involves the oxidation of diphenolic groups of dopamine, followed by its polymerization into polydopamine using the enzyme laccase. Lastly, the electropolymerization method has mainly been used for the formation of polydopamine on an electrode. In a deoxygenated solution, a polymeric film with high thickness can be effectively obtained by applying an appropriate electrical voltage. One disadvantage of this method, however, is the requirement that the surface of the electrode is conductive, hence polydopamine can only be deposited on conductive materials.

Despite a number of attempts to mimic natural melanins, synthetic melanins often have altered structural and functional properties compared to natural melanins (Ligonzo et al. 2009; Bridelli and Crippa 2010). Some reports have demonstrated that natural melanins in biotechnological applications outperform synthetic melanins. For instance, sepia melanin exhibits a higher specific capacity (16.1 in comparison with 7.9 mAhg^−1^) in aqueous sodium-ion batteries compared to polydopamine (Kim et al. 2013). The higher efficiency of natural melanins may be attributed to their innate features, including the carboxyl content of the starting precursor (i.e. tyrosine or L-DOPA vs. dopamine, which is devoid of a carboxylic group), the melanogenesis mechanism (Pezzella et al. 1997), the unique nanostructure of melanin granules attached to small amounts of proteins, and the higher hydration degree of the molecules (Bernsmann et al. 2010).

## Melanin production by microorganisms

The diverse biological roles of melanin in bacteria and fungi have been extensively reported in the literature (Nosanchuk and Casadevall 2003; Plonka and Grabacka 2006; Eisenman and Casadevall 2012; Solano 2014; Cordero and Casadevall 2017). However, the control of melanin synthesis in different microorganisms has only recently been investigated. Considering the advantages of using microorganisms to produce melanin, such as no seasonal growth constrains, cost-effectiveness, and eco-friendliness, microbial melanin is a valuable source of natural melanin.

**Fig. 2:**
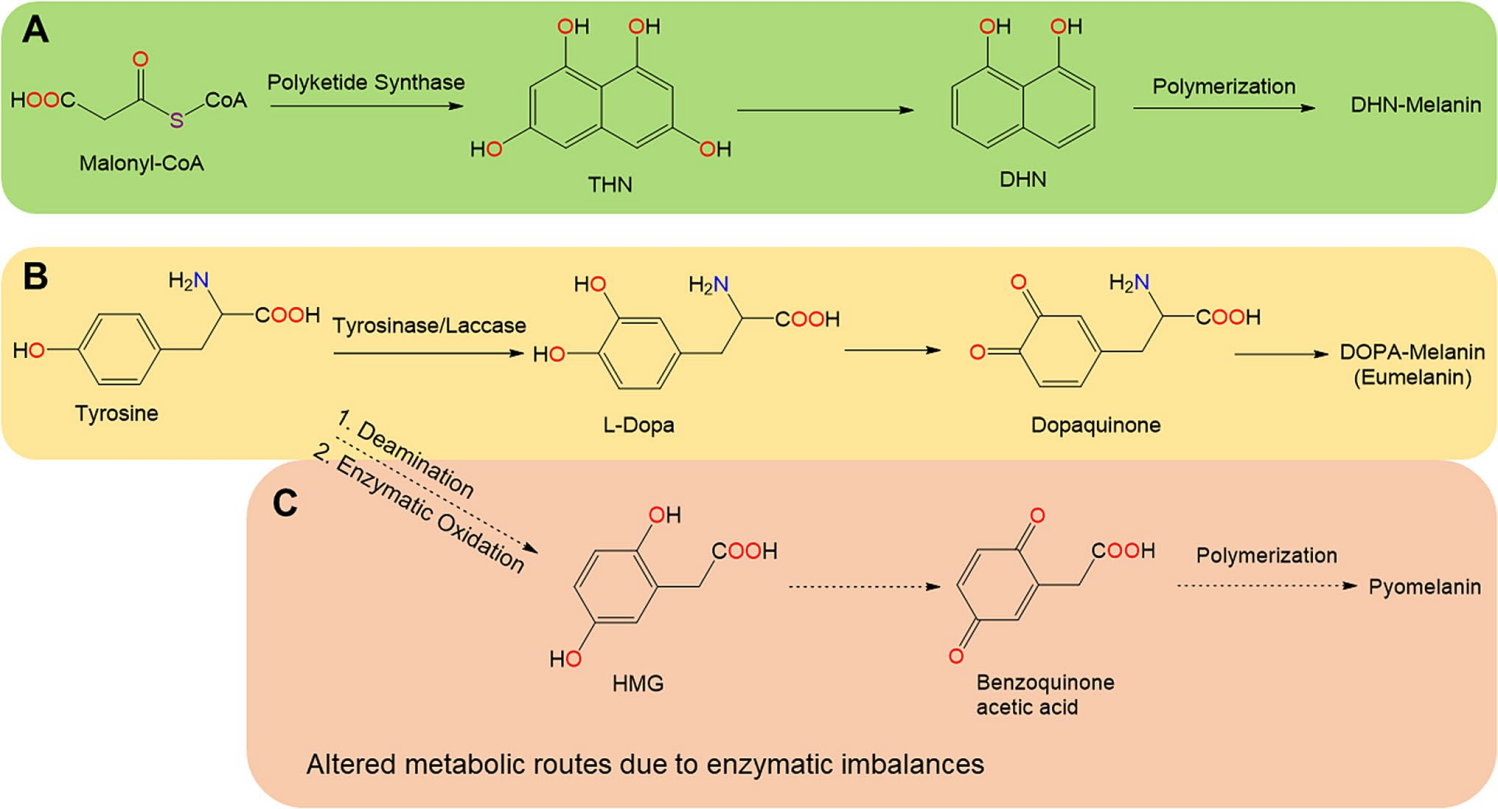
Schematic representation of melanin synthesis in bacteria and fungi, indicating key chemical transformations common to microbial melanin forming processes. **a** DHN-pathway; **b** DOPA-pathway; **c** In the event of enzymatic imbalances, altered metabolic pathway can occur, leading to different types of melanins, for example pyomelanin

Generally, most microbial melanins are formed through the transformation of either tyrosine (DOPA-pathway) or malonyl-coenzyme A (DHN-pathway), facilitated by different sets of enzymes (Fig. 2). The first pathway is very similar to mammalian melanin synthesis. In this pathway, the melanin precursor, tyrosine, is converted to L-Dopa, then to dopaquinone by tyrosinase and laccase. Dopaquinones are highly active and spontaneously oxidized and autopolymerized to form melanin. Synthesis of melanin via the DOPA-pathway is referred to as DOPA-melanin or eumelanin. However, during the catabolic process, other hydroxylated aromatic compounds such as homogentisic acid, can accumulate due to enzymatic imbalances or interruptions, which may result in different types of melanins. In the second pathway, the corresponding precursor, malonyl-coenzyme A, is produced endogenously. Catalyzed by polyketide synthases, the sequential decarboxylative condensation of five molecules of malonyl-coenzyme A creates 1,3,6,8-tetrahydroxynaphthalene (THN). THN then undergoes a series of reduction and dehydration reactions to form 1,8-dihydroxynaphthalene (DHN). The polymerization of DHN results in DHN-melanin as the final product (Plonka and Grabacka 2006; Eisenman and Casadevall 2012; Pavan et al. 2020). Notably, both pathways can be found in bacteria and fungi. Nevertheless, most bacteria and basidiomycetous fungi synthesize melanin via the DOPA-pathway, whereas, ascomycetous and some imperfect fungi including non-microscopic fungi, for instance *Tuber* spp., use the DHN-pathway to produce melanin.

With respect to high-yield melanin production, microorganisms using the DHN-pathway are not preferred since in this pathway, the pigment is synthesized endogenously and is tightly attached to the inner side of the cell wall (Toledo et al. 2017). This makes melanin extraction extremely difficult and can generate artifacts derived from harsh extracting chemicals. Alternatively, melanogenesis via the DOPA-pathway is a mechanism microorganisms use to neutralize toxic phenolic compounds from the environment, such as those released by microorganisms during host defense (Schmaler-Ripcke et al. 2009; Almeida-Paes et al. 2012). Consequently, many microbes depend on exogenous tyrosine or tyrosine derivative substrates for melanin synthesis. This is of great interest for scientists that study microbial melanization since melanin is produced extracellularly and harsh extraction can therefore be avoided. Several bacteria and fungi in this category are summarized in Table 2.

**Table 2:** Studies focused on optimization of microbial melanin production

Microorganisms	Melanin type	Max. melanin production/g L^−1^, (Incubation time / days)	Tyrosine added	Metal ions added	Substrates	References
Bacteria						
* Actinoalloteichus* sp. MA-32	DOPA	0.1 (7)	Yes	Fe, Mg	Glycerol	(Manivasagan et al. 2013)
* Bacillus safensis*	Nd	6.9 (24 h)	None	None	Fruit waste extract	(Tarangini and Mishra 2014)
* Brevundimonas* sp. SGJ	DOPA	6.8 (54 h)	Yes	Cu	Tryptone	(Surwase et al. 2013)
* Nocardiopsis alba* MSA10	Nd	3.4 (7)	Yes	Nd	Sucrose	(Kiran et al. 2014)
* Pseudomonas* sp. WH001 55	Nd	7.6 (6)	Yes	None	Starch, yeast extract	(Kiran et al. 2017)
* Pseudomonas stutzeri* HMGM-7	DOPA	7.2 (3)	Yes	None	Nutrient broth in sea water	(Ganesh Kumar et al. 2013)
* Streptomyces glaucescens* NEAE-H	DOPA	0.4 (6)	Yes	Fe	Protease peptone	(El-Naggar and El-Ewasy 2017)
* Streptomyces kathirae* SC-1	DOPA	13.7 (5)	Yes	Cu	Amylodextrine, yeast extract	(Guo et al. 2014)
* Streptomyces lusitanus* DMZ-3	nd	5.3 (6)	Yes	Cu	Beef extract	(Madhusudhan et al. 2014)
* Streptomyces* sp. ZL-24	DOPA	4.2 (5)	None	Fe, Ni	Soy peptone	(Wang et al. 2019)
Fungi						
* Armillaria borealis*	DOPA	11.58 (97)	Yes	Cu, Fe, Mg	Glucose, yeast extract	(Ribera et al. 2019)
* Armillaria cepistipes*	DOPA	27.98 (161)	Yes	Cu, Fe, Mg	Glucose, yeast extract	(Ribera et al. 2019)
* Armillaria ostoyae*	DOPA	24.80 (153)	Yes	Cu, Fe, Mg	Glucose, yeast extract	(Ribera et al. 2019)
* Aspergillus fumigatus*	Nd	0.01 (10)	No	None	Dextrose, peptone	(Raman et al. 2015)
* Auricularia auricula*	DOPA	2.97 (8)	Yes	Mg	Lactose, yeast extract	(Sun et al. 2016)
* Daldinia concentrica*	DOPA	1.78 (73)	Yes	Cu, Fe, Mg	Glucose, yeast extract	(Ribera et al. 2019)
* Gliocephalotrichum simplex*	DOPA	6.60 (6)	Yes	Cu, Fe	Peptone, yeast extract	(Jalmi et al. 2012)

*Nd* not defined

Additionally, these characteristics allow considerable control of the yield and the type of resulting melanin. Although tyrosine is identified as the main melanin substrate, other catecholamines such as dopamine and norepinephrine can also be used as substrates. However, it is important to note that melanins resulting from different substrates may differ in structure due to various catabolic processes with different enzymes involved. This creates room for tuning the physicochemical properties and optimizing the production of microbial melanins.

The formation of melanin depends highly on the regulation of melanin synthesis enzymes, which is driven by multiple nutritional factors and physicochemical conditions. Peptone, glucose and yeast extract are widely chosen as carbon and nitrogen sources. Recent studies have also exploited agricultural residues, such as fruit waste extract, corn steep liquor and wheat bran extract, to lower the production cost while ensuring the high yield of production (Hamano and Kilikian 2006; Silveira et al. 2008; Zou and Tian 2017). Copper is an important element for melanin production because of its role as a cofactor for laccases and tyrosinases (Sendovski et al. 2011; Reiss et al. 2013; Yang et al. 2017). Variation in the amount of added copper leads to irregular pigmentation in several fungal and bacterial species (Held and Kutzner 1990; Griffith et al. 2007). On the one hand, besides copper, other metals can also enhance melanin formation. A recent study by Wang et al. (2019) showed a strong increase in tyrosinase activity and melanin production driven by the addition of iron and nickel. On the other hand, the presence of metals may induce stress responses in microbes, resulting in melanin formation (Gowri and Srivastava 1996). In other cases, melanin synthesis is promoted by different kinds of stress, for instance: high temperature, nutrient-poor growth media, hyperosmotic pressure, etc. (Coyne and Al-Harthi 1992; Fogarty and Tobin 1996; Cordero and Casadevall 2017). Because of the multiple and diverse factors that affect melanin biosynthesis, there is no universal culture media or cultivation condition for growing melanogenic microorganisms. Instead, the composition and ratio of each component should be identified depending on the microbe. Similarly, environmental factors, i.e. temperature, pH, the presence of oxygen and aeration, light, stress and irradiation during cultivation, can greatly affect the cell growth and pigment biosynthesis, and should be carefully considered. Some statistic tools such as the Taguchi method, the Plackett-Burman design, or the Response surface methodology are usually used to design multifactorial experiments and to evaluate the impact of each factor in the production process (Surwase et al. 2013; Saini and Melo 2015; Sun et al. 2016; El-Naggar and El-Ewasy 2017).

Previously, most melanin-related studies involving other microorganisms, such as *Aspergillus carbonarius* or *Streptomyces glaucescens* NEAE-H (El-Naggar and El-Ewasy 2017), could not achieve melanin suitable for industrial application yields (< 1 g L^−1^ medium) even after optimizing the growth conditions. In contrast, studies focused on optimizing melanin production utilize fungi and bacteria with the ability to produce melanin via tyrosine transformation. The optimized protocols usually involve the exogenous supply of tyrosine, copper and/or other metal ions and show the possibility to produce melanin pigments in significant yields. For instance, melanin can now be produced by *Armillaria cepistipes* at gram-scale (28 g L^−1^ medium) in laboratory condition, which paves the way for industrial scaling up and future applications of melanin (Ribera et al. 2019).

Last but not least, microbial melanin production can be further improved by applying genetic engineering techniques to increase the natural melanogenic capacity of some organisms or generating novel melanin-producing strains. The most common genetic modification to enhance/generate a production strain, targets the expression of genes encoding the enzymes involved in melanin formation, mostly tyrosinases. The latest advances in the generation of recombinant melanogenic strains and production processes were already summarized by (Martínez et al. 2019) and are not the focus of this review.

## Applications of microbial melanins

**Fig. 3:**
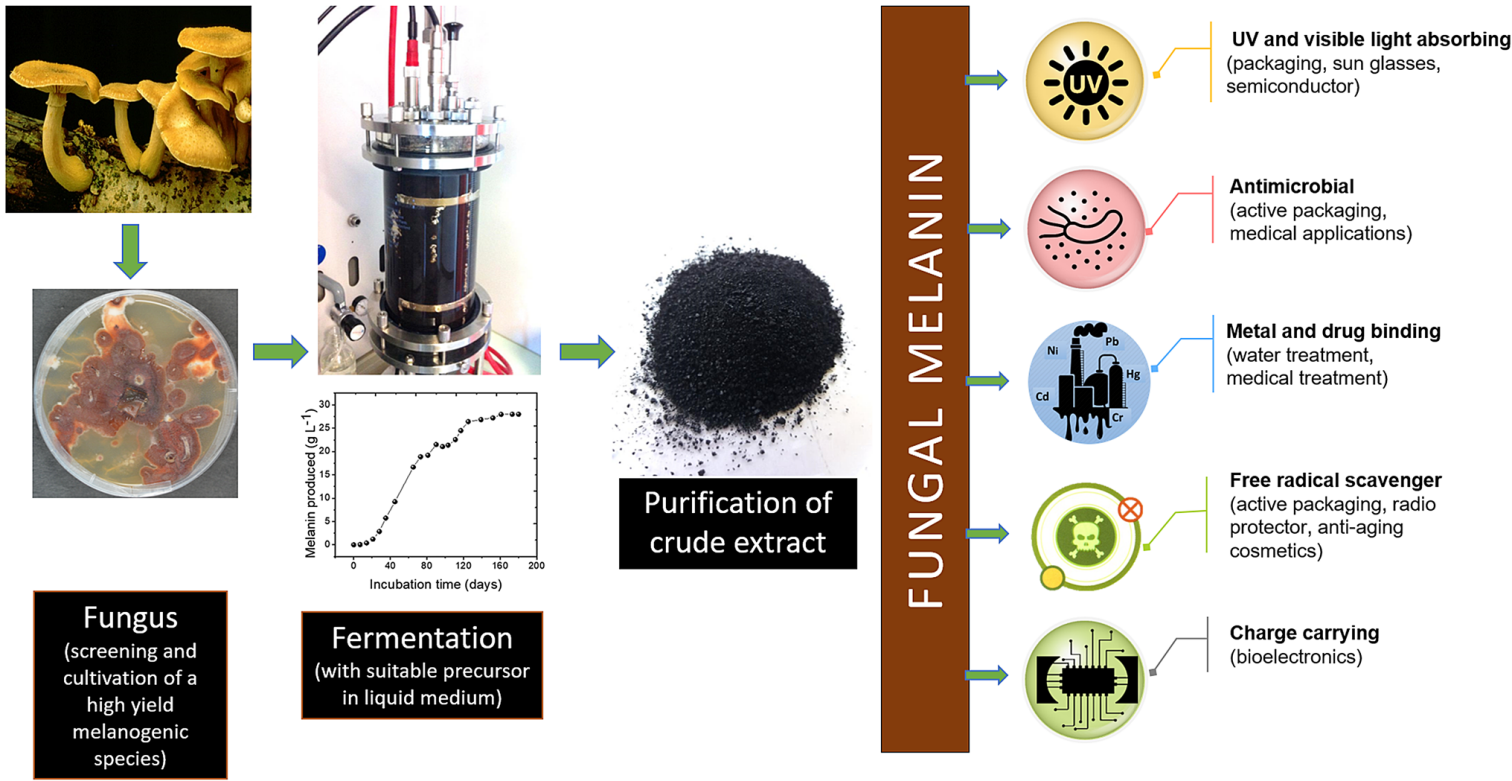
Biotechnological production of fungal melanin and its potential applications

In fungi and bacteria, melanins are usually reported for its important role in the virulence of pathogenic organisms (Jacobson 2000; Cordero and Casadevall 2017). With the advancement of new knowledge and technologies, melanin pigments can now be turned into valuable materials in various fields of green technology, materials science, biomedicine, cosmetics and environmental remediation (Fig. 3).

From a physicochemical viewpoint, melanin is a natural “sunscreen” that absorbs the broadband of UV–visible light spectrum. In addition to blocking UV light, this pigment is a powerful antioxidant. Melanin also exhibits a hydration-dependent semiconductor-like behavior. As such, it is evaluated as a component for organic electronic devices (Bothma et al. 2008; Kim et al. 2013). Other advantages of microbial melanin are its bioavailability, biocompatibility and biodegradability, making it a promising candidate for biomedical applications; for example, implantable devices (Vahidzadeh et al. 2018). In another type of application, melanin has been employed for the environmentally benign synthesis of silver nanostructures. These melanin-mediated silver nanostructures show broad-spectrum antimicrobial activity against food pathogens and have potential uses in the food and health industries (Kiran et al. 2014; Patil et al. 2018). Dermal and cosmetic applications of melanin include its use for sunscreen and hair dyeing. Melanin can act as metal chelators, which can be employed in environmental applications. By incorporating fungal melanin with other polymers such as polycaprolactone and polyurethane, the melanin-based composites can remove up to 94% Pb(II) in water systems (Tran-Ly et al. 2020). Although there are a lot of studies reporting the potential applications of melanin, they are mostly in the developmental stage and have not yet been commercialized. In the era of transition towards sustainable materials, microbial melanin research has not yet reached its full potential.

## Conclusions and outlook

Melanins are a unique class of natural pigments that can be considered functional materials for multiple potential applications in industry. The future of melanin-based materials and technology development depends on the ability to produce melanin at a large scale with a chemically defined structure and low cost. As discussed in this review, conventional approaches are the isolation of melanins from natural sources like sepia ink and chemical synthesis. They are, however, unsustainable and difficult to scale up. A feasible alternative approach is using melanogenic microorganisms and melanin precursors. Although no universal protocol is available, good tips for producing high-yield microbial melanins are: (i) choosing the microorganisms that can produce melanin extracellularly from the exogenous substrate, and (ii) improving the metabolic process by adding tyrosine and copper to the culture media. However, it is worth pointing out that melanins comprise a chemically-diverse group of polymers. So far, most of the published works on microbial melanin production focused on eumelanin. However, the chemical diversity of melanin, which can be controlled by the supplied melanin precursors, has not yet been fully explored. Furthermore, the accumulated knowledge on the biochemistry and genetic engineering of melanin in various organisms can contribute to the direct manipulation and enhancement of melanin production. With this perspective, melanin can be used beyond basic research and encourage more researchers from industry to deploy bio-inspired melanin-based materials for biomedical, environmental and technological applications.
